# Conductance of a single flexible molecular wire composed of alternating donor and acceptor units

**DOI:** 10.1038/ncomms8397

**Published:** 2015-07-06

**Authors:** Christophe Nacci, Francisco Ample, David Bleger, Stefan Hecht, Christian Joachim, Leonhard Grill

**Affiliations:** 1 Department of Physical Chemistry, Fritz-Haber-Institute of the Max-Planck-Society, Berlin 14195, Germany..; 2 Department of Physical Chemistry, University of Graz, Graz 8010, Austria.; 3 Institute of Materials Research and Engineering (IMRE), Singapore 117602, Singapore.; 4 Department of Chemistry and IRIS Adlershof, Humboldt-Universität zu Berlin, Berlin 12489, Germany.; 5 Nanosciences Group and MANA Satellite, CEMES-CNRS, Toulouse 31055, France.; 6 International Center for Materials Nanoarchitectonics (MANA), National Institute for Materials Science (NIMS), 1-1 Namiki, Tsukuba, Ibaraki 305-0044, Japan.

## Abstract

Molecular-scale electronics is mainly concerned by understanding charge transport through individual molecules. A key issue here is the charge transport capability through a single—typically linear—molecule, characterized by the current decay with increasing length. To improve the conductance of individual polymers, molecular design often either involves the use of rigid ribbon/ladder-type structures, thereby sacrificing for flexibility of the molecular wire, or a zero band gap, typically associated with chemical instability. Here we show that a conjugated polymer composed of alternating donor and acceptor repeat units, synthesized directly by an on-surface polymerization, exhibits a very high conductance while maintaining both its flexible structure and a finite band gap. Importantly, electronic delocalization along the wire does not seem to be necessary as proven by spatial mapping of the electronic states along individual molecular wires. Our approach should facilitate the realization of flexible ‘soft' molecular-scale circuitry, for example, on bendable substrates.

## Abstract (web summary)

Molecular wires with high conductivity and conformable flexibility are desirable in nanoelectronics. Here, Nacci *et al.* grow flexible molecular wires, composed of alternating donor and acceptor units, on a metal surface, and show that high conductance can be achieved without electronic delocalization.

## Graphical abstract

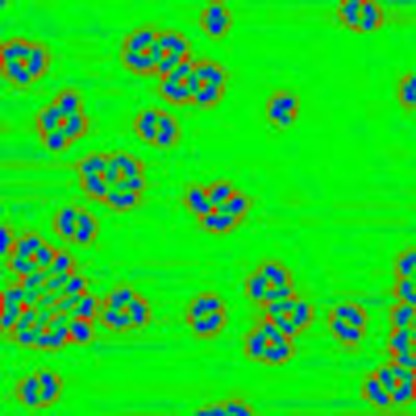

Molecular wires are key elements in nanoelectronics, in particular in single-molecule circuitry where the wires are responsible for electronic communication between various functional elements^1^,^2^. The important criteria for an optimal molecular wire are efficient charge transport, high chemical stability and a high conformational flexibility to adapt to different contact geometries. In this regard, it is of utmost importance to study and understand charge transport through a *single* molecular chain and to correlate it with its specific chemical structure and associated electronic properties in order to optimize its conductance. Back in 1974, Aviram and Ratner^3^ have theoretically proposed to study long-range electron transfer processes through a single molecule composed of an electron-donating subunit covalently connected via a bridge to an electron-accepting subunit, thereby conceiving a molecular rectifier. However, realizing their vision and exploring charge transfer at the level of a single molecule is a challenging task that continues to attract considerable attention, both from theory^4^ and experiment^5^. Electron transfer through a molecular junction depends on many parameters: the chemical composition of the molecule^6^, its conformation^7^,^8^ and the electrode contacts^9^,^10^,^11^ as well as the energy of the electrons that pass through the molecule^12^.

The measurement of single-molecule conductance is experimentally difficult because one needs to bridge various orders of magnitude from the macroscopic electrodes to the molecular wire at the nanometre scale. Several methods have been developed in the last years, which can be essentially divided into two categories: on one hand statistical methods^6^,^7^,^13^,^14^,^15^,^16^ and on the other hand non-statistical single-molecule pulling approaches^12^,^17^,^18^,^19^. Assuming the validity of the standard Kirchhoff circuit mesh law^20^, the former allows to explore the conductance of a molecular junction made of an ensemble of molecules located within a two-electrode junction, for instance, using a mechanical break junction set-up, and the properties of a single molecule are then obtained from a statistical analysis. This fast method allows performing many experiments but offers limited insight about the atomic-scale structure, the exact number of active molecules or the conformation of each molecule in the junction. The latter method, pulling a single molecular wire with the tip apex of a scanning tunnelling microscope (STM) at low temperatures, is slower and therefore provides much less data in a reasonable time frame. However, it includes precise information on the single molecular junction environment since one can select a well-defined molecular wire on the surface by STM imaging before (and typically also after) the conductance measurement. Furthermore, it allows to measure the conductance directly during pulling as a function of the molecular chain length, which is the key property of molecular wires, in contrast to the comparison of modified set-ups with different molecules of various lengths in statistical experiments^6^.

In order to design an ideal molecular wire with an optimal charge transport, a small (but not zero) highest occupied molecular orbital (HOMO)– lowest unoccupied molecular orbital (LUMO) gap *E*_g_ (at fixed chain length) is typically desired. Two main strategies have been followed over the years^21^. On one hand, effective *π-*conjugation has been enforced by ladder-type structures, such as oligoacenes^22^, porphyrin tapes^23^ and recently in graphene nanoribbons^12^, which avoid bond-length alternation due to Peierl's distortion^24^; however, the rigidity of these molecular wires limits their use in flexible set-ups requiring the wire to adapt to surface/substrate bending. On the other hand, coupling electron donor and acceptor units via *π*-conjugated bridges leads to a significant lowering of *E*_g_, as has been known for dyes for a long time and proven to be a viable strategy for developing organic optoelectronic materials^25^,^26^. In the context of single molecular wire conductance measurements the first approach suffers from the necessity of a post-functionalization step to annulate the initially formed polymer precursor to a rigid ribbon^12^,^27^, whereas the latter approach offers the benefit of generating a polymer with an inherently low *E*_g_ yet maintaining a somewhat flexible structure. Keeping the *E*_g_ of the molecular wire finite is beneficial to maintain its chemical stability, which is typically lowered by oxidation because of decreasing ionization potentials, that is, high-lying HOMO levels. Note that at zero band gap a high conductance has been determined in statistical measurements^28^. The conductance of polymers with alternating donor and acceptor units has been studied^29^; however, many molecules (the exact number of molecules has been estimated to be ∼100) are present in the junction at the same time^6^ and the conductance of molecules in an ensemble can differ significantly from a single molecule^30^. Moreover, the conductance values are potentially shifted by undesired charge carriers because of light from the microscope set-up^29^.

In analogy to inorganic semiconductors and insulators, where the incorporation of foreign atoms (dopants) into their crystals^31^ is an effective strategy to modify their band structure by introducing new states for electrons and/or holes^32^, we utilize the donor and acceptor units to lower the band gap of the polymer chain^25^. Here we show that on-surface polymerization can be used to generate flexible molecular wires, composed of alternating donor and acceptor units, which despite the lack of electronic delocalization along the chain and a non-zero *E*_g_ exhibit a high conductance around the Fermi level in single-molecule transport measurements.

## Results

### On-surface polymerization

**Figure 1:**
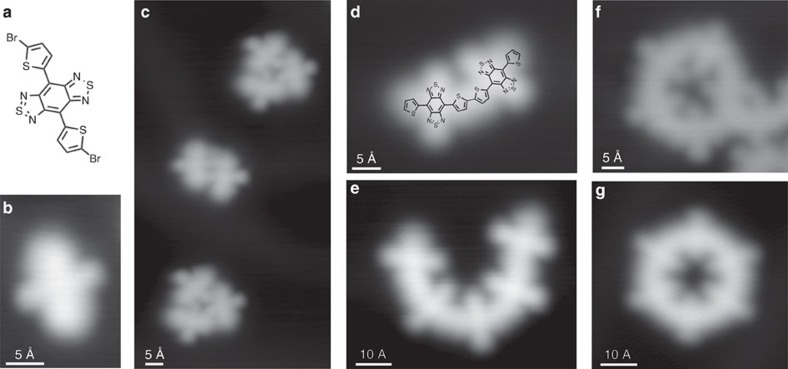
DAD molecules and polymers on an Au(111) surface. (**a**) Chemical structure of the Br-DAD-Br molecule and (**b,c**) molecules on an Au(111) surface before polymerization. (**b–g**) STM images of various molecular structures (overview STM images are presented in Supplementary Fig. 1). (**b**) The two lateral sharp round features of a single Br-DAD-Br (16 Å × 23 Å) molecule are associated to the acceptor sites (thiadiazole rings in **a**). (**c**) At larger scale (55 Å × 103 Å), agglomerates comprising few Br-DAD-Br molecules are identified. Set point: 0.5 V, 100 pA. Note that the position of the sulfur atoms at the outer thiophene rings is estimated in the superimposed chemical structure while that of the inner ones is deduced from the oligomer geometry (Supplementary Fig. 2). (**d**–**g**) After polymerization. (**d**) STM image of a (DAD)_2_ chain (40 Å × 30 Å) and (**e**) (DAD)_5_ chain (64 Å × 53 Å), set point: 1 V, 50 pA. Closed structures are clearly identified at the surface: (a) (DAD)_5_ ring is shown in **f** (43 Å × 38 Å, 0.5 V, 100 pA) and a (DAD)_6_ ring is shown in **g** (53 Å × 51 Å, 1 V, 50 pA). (DAD)_6_ is the most abundant closed structure (95%), the rest is just represented by (DAD)_5_ rings.

The target polymer structure was inspired by recent lines of work on alternating donor–acceptor polymers in the context of organic solar cells^25^,^33^. In particular, the combination of alternating oligothiophene donors and benzobis(1,2,5-thiadiazole) acceptor units, which in addition facilitate the formation of quinoid structures, has proven to lower the HOMO–LUMO gaps far below 1 eV (ref. 34). Very recently, Fasel and co-workers^35^ have used on-surface synthesis to prepare a molecular chain with a random mixture of undoped and doped graphene nanoribbon sections, thus creating characteristic electronic transitions between them in analogy to p–n junctions. However, to obtain a well-defined alternation of donor and acceptor units, on-surface polymerization involving step growth via dehalogenative Ullmann-type coupling reactions requires the integration of both donor and acceptor units into one symmetrical monomer structure carrying two reactive halide termini and hence we prepared bis(5-bromo-2-thienyl)-benzobis(1,2,5-thiadiazole) as the Br-DAD-Br monomer (Fig. 1a). The abbreviation DAD stems from the alternation of donor (D) and acceptor (A) groups, namely an (acceptor) central benzobis(thiadiazole) unit carrying two lateral (donor) thiophene rings.

When depositing the Br-DAD-Br molecules (Fig. 1a) on a Au(111) surface, STM images of single molecules show an elongated bright protrusion with two smaller protrusions at the centre (Fig. 1b,c). From their unique appearance in combination with image calculations (Supplementary Figs 3 and 4), we can assign the different features to chemical groups within individual DAD molecules. The Br substituents cause the bright protrusions at the termini, while the outer molecular sites across the elongated direction are associated with the thiadiazole groups. When inducing dehalogenative coupling reactions between the Br-DAD-Br monomers on the surface by heating at 520 K, (DAD)_*n*_ oligomers with covalent bonds between the DAD units^36^ are formed (Fig. 1d,g). Interestingly, we find not only linear chains of different lengths that are characteristic for this process (Fig. 1d,e), but also closed rings (Fig. 1f,g) similar to cyclooligothiophenes^37^. (DAD)_6_, which is the most abundant closed structure, has a curvature radius of ∼13.9±0.2 Å while (DAD)_5_ exhibits 11.5±0.2 Å (Supplementary Fig. 5), indicating a high degree of conformational flexibility of the DAD chains. All oligomers on the surface reveal high structural regularity. Their characteristic shape is in agreement with our molecular design and proves the successful polymerization process, leading to the desired molecular wires composed of benzobis(1,2,5-thiadiazole) moieties linked via flexible bithiophene units.

### Electronic structure of DAD polymers

**Figure 2:**
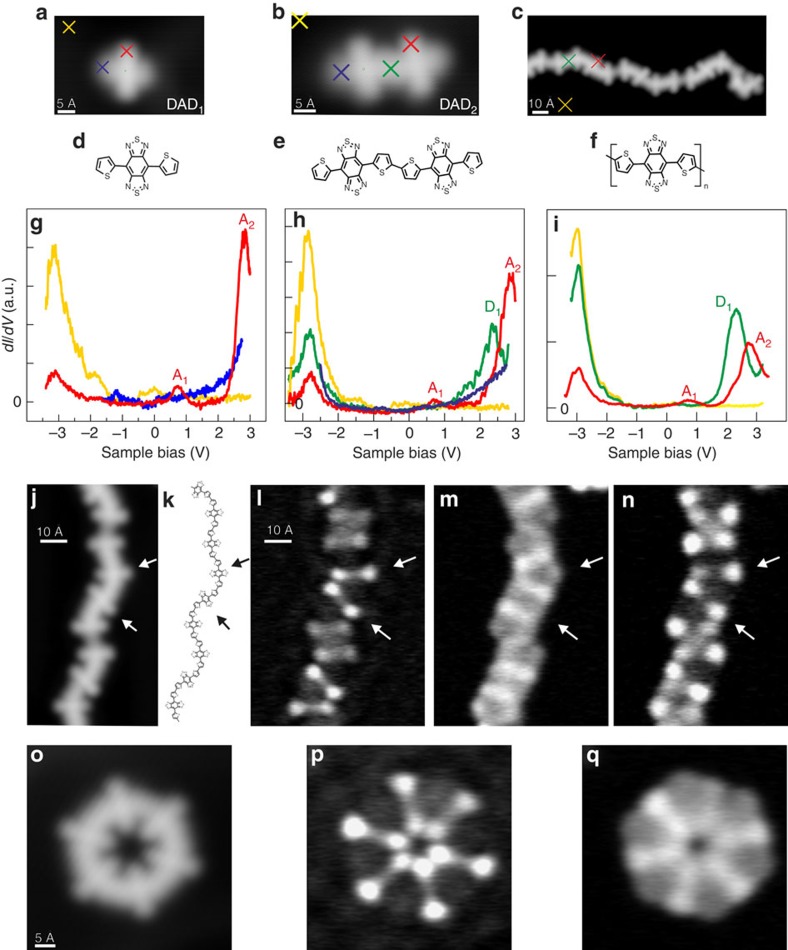
Comparing the electronic structure of monomers and different polymers. Differential d*I*/d*V* conductance spectra taken above a single dehalogenated DAD monomer (**a**,**d**,**g**), (DAD)_2_ dimer (**b**,**e**,**h**) and (DAD)_18_ chain (**c**,**f**,**i**). Two molecular states are identified when probed at the acceptor sites (**g–i**, red curves), accordingly denominated A_1_ and A_2_. A new molecular state is identified in the middle of the dimer (**h**, green curve), that is, thiophene dimer site, at ∼2.3 eV (D_1_). d*I*/d*V* mapping of donor- and acceptor-like molecular states. Open structures: (**j**) STM image (49 Å × 90.5 Å, 0.5 V, 100 pA) of a chain segment, the chemical structure (**k**; see Supplementary Fig. 2 how the conformation is assigned), and d*I*/d*V* maps (65.5 Å × 88.5 Å, constant-current mode) at energies corresponding to its molecular states: (**l**) 0.75 V (A_1_), (**m**) 2.3 V (D_1_) and (**n**) 2.76 V (A_2_). The arrows indicate two adjacent acceptor sites. Closed structures: (**o**) STM image of a (DAD)_6_ ring (50 Å × 50 Å, 1 V, 50 pA) and conductance maps of (DAD)_6_ (50 Å × 50 Å) taken at energies of 2.15 V (**p**) and 2.73 V (**q**), D_1_ and A_2_, respectively (for details see Supplementary Fig. 6).

In order to gain insight into electronic delocalization along the molecular wires, the intramolecular electronic structure of single chains was spatially resolved using low-temperature scanning tunnelling spectroscopy (STS). In particular, the development of the electronic states as a function of the (DAD)_*n*_ length was measured by comparing STS peak positions for different chains. Figure 2a shows the d*I*/d*V* spectra of an activated (that is, after Br dissociation) individual DAD monomer. By placing the STM tip at different positions over the molecule (and over the pure gold surface as a reference to eliminate contributions from the tip apex and the metal surface states), the d*I*/d*V* spectrum at the acceptor location (that is, the benzobis(1,2,5-thiadiazole) group) reveals the presence of two clear features, one at low energy (A_1_, at ∼0.8 eV) and one at higher energy (A_2_, at ∼3 eV), respectively. At the thiophene (that is, donor) locations, the d*I*/d*V* spectrum increases monotonically (at positive bias) without showing any particular feature (at least, up to 3.2 eV). At negative bias, electronic resonances cannot be clearly identified in STS because the HOMO states of the DAD monomer are not very well coupled to the Au(111) surface as we found in our calculations (Supplementary Fig. 3). This comes from the fact that the molecular wires are physisorbed and the spatially more extended LUMO orbital overlaps better with the surface.

**Figure 3:**
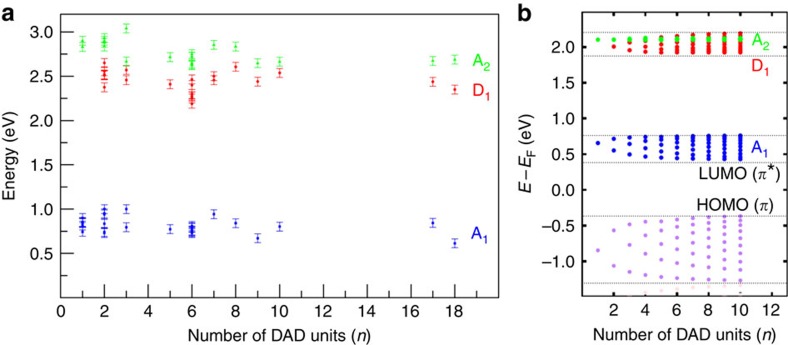
Electronic states for different polymer lengths. (**a**) Energy position of states A_1,2_ and D_1_ measured by d*I*/d*V* spectroscopy as a function of the DAD oligomer length. The error bars reflect the precision in the determination of the peak maxima in d*I*/d*V* spectra. (**b**) Calculated energy dispersion of the (DAD)_*n*_ monoelectronic states as a function of the number of units (see Supplementary Fig. 7 for details). The small dispersion of the A_1_, D_1_ bands and the zero dispersion of the A_2_ band with the corresponding accumulation of its electronic states as a function of the number of unit within each band width can be seen. The calculated HOMO state dispersion is also represented as a reference.

To understand the localization of the electronic states along the oligomers, it is necessary to investigate the evolution of the molecular states with the oligomer length, similar to the studies of homogeneous thiophene structures^37^,^38^. When comparing the monomer with a dimer ((DAD)_2_; Fig. 2b), we find a new state at ∼2.3 eV (D_1_) at the donor location, in agreement with our calculated spectra (Supplementary Fig. 4) while the acceptors states A_1_ and A_2_ are left undisturbed (note that all these are unoccupied states). Interestingly, it is spatially located at the site of the bithiophene within the molecular chain but no peak is visible at this energy position at the thiophene site for the DAD monomer (Fig. 2a) or at the terminus of a chain. Thus, the D_1_ state is clearly related to the linking of two thiophenes to a bithiophene unit, which is the actual donor moiety in the formed DAD polymer. Such an effect on the optoelectronic properties (via the energy positions of the electronic states) with increasing chain length is well established in the field of *π*-conjugated oligomers^39^. For longer (DAD)_*n*_ chains, the d*I*/d*V* spectra show the persistence of these three states A_1_, A_2_ and D_1_ without any significant energy shift (Fig. 2i). This is also valid for longer (DAD)_*n*_ chains with up to *n*=18, corresponding to a length of ∼23 nm (see Fig. 3), and in agreement with a very small energy dispersion (lower than 0.2 eV) in the calculated electronic structure (Fig. 3b and Supplementary Fig. 7). Hence, these results indicate that polymerization leads only to a rather small electron delocalization along the molecular chain.

In order to measure the spatial distribution of these molecular states across the wire structure, we have determined conductance maps where the d*I*/d*V* signal over a chosen surface area is plotted for a given bias voltage^40^,^41^, thereby spatially resolving the electronic structure along a single polymer. At bias voltages corresponding to states A_1_ and A_2_ (0.75 and 2.76 V, respectively), the conductance maps of a chain reveal a dumbbell-like pattern (Fig. 2l,n); thus, a clear localization of these states at the centres of the molecular building blocks, each of them giving rise to a double lobe. Hence, these states are localized at the acceptor sites as visible in Fig. 2a,b. On the other hand, the d*I*/d*V* map at energies corresponding to the donor state D_1_ (Fig. 2m) shows an enhanced signal intensity at the donor sites, resulting in an inverted pattern compared with the previous one (although the background intensity from peak A_2_ is still visible as can be understood from Fig. 2i). The same is valid for closed loop structures (see Fig. 2o–q and, with more details, Supplementary Fig. 6). This shows that also the donor state is very localized and that, in agreement with the interpretation above, the polymer's electronic states at small energies exhibit only a very weak electronic delocalization.

### Theoretical description

After characterizing the electronic structure of DAD chains when adsorbed flat on the Au(111) surface, electron transport was studied by pulling single oligomers with the STM tip off the surface^18^. For a given bias voltage *V* and in first approximation when *T*(*E*) is not changing too much with *V* (ref. 42), the current intensity *I*(*L*,*V*) passing through a single molecular wire junction is given by


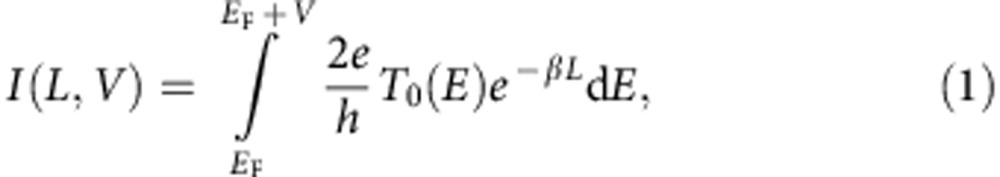


with 2*e*^2^/*h T*_o_(*E*) being the electron energy-dependent contact conductance. *L* is the length of the molecular wire and *β*(*E*) the energy-dependent tunnel inverse decay length through the molecular wire that characterizes the conducting performance of the molecular wire. In a monoelectronic approach, *β*(*E*) depends on the effective mass m*(*E*) of the tunnelling electrons, on the HOMO–LUMO gap *E*_g_, and on the relative position of the HOMO and LUMO electronic states relative to the Fermi level *E*_F_ (refs 12, 18, 19, 43). Accordingly, the conductance significantly increases as soon as the electron energy matches the energy of a molecular electronic state (within the HOMO–LUMO gap *β*(*E*) remains non-zero), which has been confirmed experimentally^12^. For an ideal fully delocalized electronic structure of the molecular wire, without relaxation of its chemical structure, the energy gap *E*_g_ and the inverse decay length *β*(*E*) both equal zero if the electronic structure of the molecular wire is matching that of the electrodes.

### Single-polymer conductance measurements

**Figure 4:**
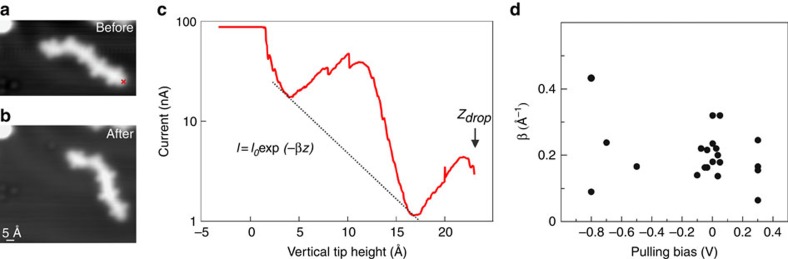
Measuring the conductance of single DAD polymers for various bias voltages. (**a**) STM image (86 Å × 48 Å, 0.5 V, 100 pA) of an individual chain comprising five DAD units. (**b**) STM image of the same area in **a** after contacting (see cross in **a**) and pulling the wire. The chain dropped down to the surface lying on a different orientation. (**c**) Tunnel current versus vertical tip displacement (pulling curve) taken at pulling bias of 50 mV (set point: 0.5 V, 100 pA). The *I*(*z*) curve has been fitted with an exponential decay relationship *I*(*z*)∝ exp(−*βz*) with *β* being the current inverse decay length factor and *z* the vertical tip displacement (black curve). The maximum current is limited to 90 nA in this case by the current pre-amplifier. *Z*_drop_ indicates the point at which the pulling failed likely because of the rupture of the bond between chain and STM tip. (**d**) *β* parameter as a function of the bias voltage applied to the STM junction while pulling at small and large bias voltages compared with the HOMO–LUMO gap of the wire. The corresponding calculations are presented in Supplementary Fig. 9. Note that the measured *β* decay factors seem to be independent of the oligomer lengths and *Z*_drop_ values (Supplementary Fig. 10).

In order to provide individual polymers for such experiments, lateral manipulation with the STM tip has been used to separate chains from each other. In the pulling experiments, the STM tip was positioned above the end of a given molecular wire, the STM feedback loop was disabled and the tip apex was then approached vertically towards the chain end until a contact was established. Afterwards, the tip was retracted and the current *I*(*z*) was measured as a continuous function of the tip height, which reflects approximately the chain length in this electrode–molecule–electrode set-up (actually it is slightly larger because of the molecular wire curvature on pulling^12^). In order to measure an electrical current through the pulled molecular wire, a bias voltage was applied between the sample and the STM tip. The *I*(*z*) pulling curve (Fig. 4) exhibits an exponential decay (with superimposed oscillations that are discussed below), typical for the low-voltage conductance variation of a molecular wire with its contacted length. Hence, the conductance of individual wires is determined via the decay constant *β* (see Equation (1)). By performing such an experiment for many equivalent molecular wires and fitting the *I*(*z*) curves (see Supplementary Fig. 8 for details), we find an inverse decay constant *β* of 0.21±0.06 Å^−1^ for bias voltages between −100 and +100 mV with respect to the Fermi level, that is, well within the (DAD)_*n*_ HOMO–LUMO gap (that is discussed below). This experimental finding is in very good agreement with the theoretical value *β*_th_=0.23 Å^−1^ in a pulling configuration (see Supplementary Fig. 9f) while we calculated a slightly smaller *β* value (0.21 Å^−1^) for a planar DAD polymer adsorbed on the surface. These are quite small *β* values in the band gap as compared with other molecular wires: 0.7–0.9 Å^−1^ for alkane^9^,^44^, 0.67 Å^−1^ for oligophenylene^45^, 0.4–0.6 Å^−1^ for graphene nanoribbons^12^, 0.4 Å^−1^ for thiophene chains^19^ and 0.3 Å^−1^ for polyfluorene^18^. Note that the *β* value is constant over the investigated oligomer lengths (Supplementary Fig. 10) and that the values measured in STM pulling are typically slightly higher^18^ because of the difference in the real polymer length in the junction and the (smaller) tip height over the surface (for example, 0.38 Å^−1^ as compared with 0.3 Å^−1^ for polyfluorene). It should be mentioned that even smaller *β* values can be achieved at small electron energies if electronic states are located close to the Fermi level, as for instance in organometallic compounds^46^; however, in the absence of such states the DAD polymer conductance is among the best determined so far.

Superimposed to the exponential decay of the current as a function of the length, we observe rather strong oscillations of the tunnelling current (Fig. 4c). These oscillations are reproducible and reveal a spatial periodicity of 11.5±1.9 Å, matching the length of an individual DAD unit in the chain, which is 13.06 Å (as determined by MM+ force field calculations). The current oscillations are caused by the detachment of one DAD unit after another from the surface while pulling the chain upwards since the effective length of the polymer is increased abruptly after each detachment, similar to polyfluorene pulling^18^ (note that the same periodicity is found in force measurements during pulling^47^). This observation is in agreement with the very high flexibility of the molecular chains, resulting in the formation of very small loops during the on-surface reaction (Fig. 1f,g and Supplementary Figs 5 and 6). The calculated *I(z)* pulling curves also show more complex oscillation structures with a periodicity of ∼11 Å (Supplementary Fig. 11). Calculations of the pulling mechanics indicate that the main (DAD)_*n*_ chain deformation occurs when an acceptor group is detached from the surface. This results in a large rotation of this group, which previously had remained flat by its adsorption on the Au(111) surface. On further pulling, two thiophene groups are then detached from the surface and they also rotate (see Supplementary Fig. 11 for details). This is in contrast to stiffer molecular chains as, for instance, graphene nanoribbons that do not exhibit any such oscillations^12^. Importantly, in the course of such a rotation new tunnelling channels can open, for example, due to a *π–σ* molecular orbital overlap during rotation, leading to an increase in the DAD conductance and therefore a rise of the measured current with the tip height (as observed in Fig. 4c).

Pulling wires at rather large bias voltages and thus at electron energies far from the Fermi level is a direct and effective way to probe the contribution of the molecular states on the wire conduction mechanism. We have measured the inverse decay constant *β* of the wire's conductance as a function of the applied bias during pulling experiments (Fig. 4d) and find—within a substantial spread (that is likely caused by the intramolecular flexibility of the DAD polymer in the junction)—an approximately constant value. Such a behaviour is very different from what has been observed for graphene nanoribbons where the decay constant dramatically drops as soon as the molecular states are matched^12^. The main difference is that the molecular states involved in the electron transport through a graphene nanoribbon were largely delocalized along the molecular chain, resulting in a large dispersion of the corresponding electronic states. Importantly, the (DAD)_*n*_ molecular wires here do not exhibit delocalized states and they are deformed during the pulling procedure, causing a step-by-step rotation of the acceptor groups (Supplementary Fig. 11). This rotation causes a shift of the electronic states towards the Fermi level and therefore a filling of the HOMO–LUMO gap (Supplementary Fig. 9), similar to the deformation of a C_60_ molecule^48^. The almost constant *β* value through a (DAD)_*n*_ chain is therefore caused by the pulling procedure and the resulting chain distortion (Supplementary Fig. 9). It is in agreement with our calculations that show constant *β* values between −0.2 and +0.2 V (Supplementary Fig. 9). This voltage range is however smaller than that in the experiment because of the underestimation of the HOMO–LUMO gap in our calculations.

### Comparing various molecular structures

**Figure 5:**
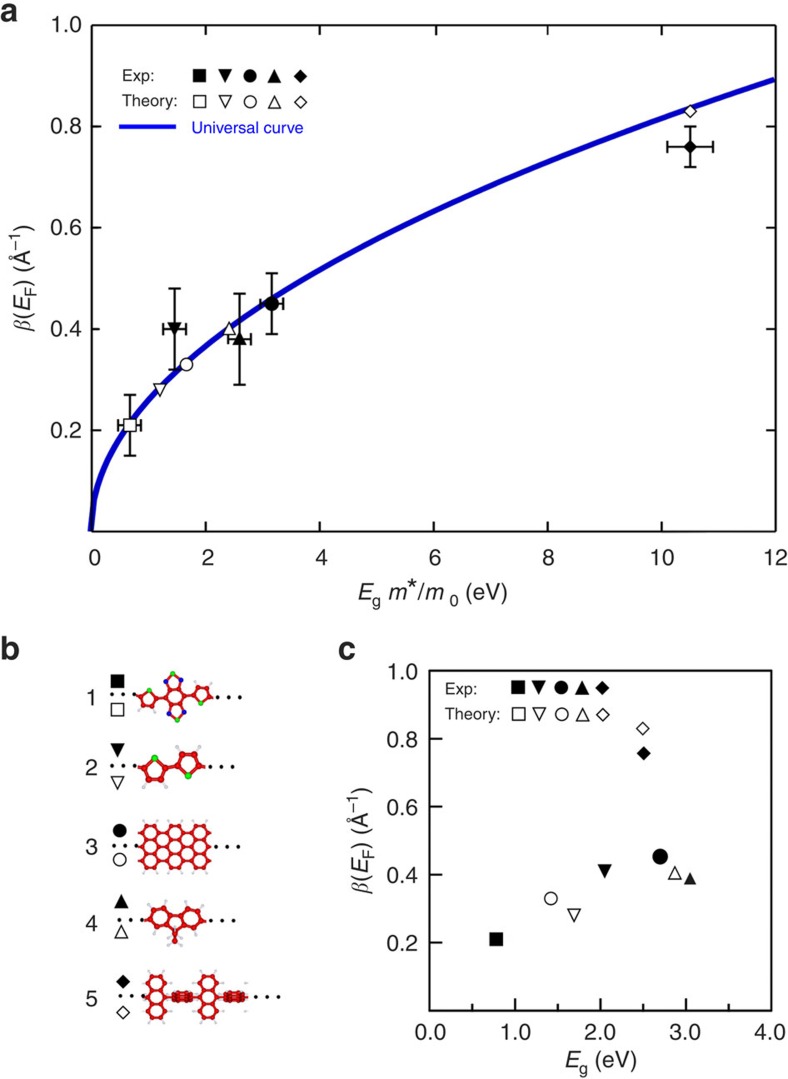
Measured and theoretical conductances of different molecular wire structures. (**a**) Experimentally determined inverse decay lengths *β* at small energies (close to the Fermi level *E*_F_) for different molecular wires (sketched in **b**) as a function of the product between the HOMO–LUMO gap *E*_g_ and the effective mass m* (*m*_o_ is the electron mass) of the tunnelling electron. All experimental *β* data points were obtained by pulling a single molecular wire in an STM junction (assuming that the tip height equals the polymer length) taken from this work (#1) and from literature for #2 (ref. 19), #3 (ref. 12), #4 (ref. 18) and #5 (ref. 12; including the error bars). The *E*_g_ values of #1 and #5 have been calculated since no experimental data are available. An estimated *E*_g_ error of ±0.4 eV is used for all data points. Note that, in absence of an experimental method to measure m*, we have done separate complex band structure calculations (which do not require values of *β*) to determine m* (and *E*_g_) independently^45^: m*/*m*_o_=0.86 (#1), 0.71 (#2), 1.17 (#3), 0.85 (#4) and 4.2 (#5; see Supplementary Fig. 13). The *β*(*E*_F_) universal curve is plotted for comparison in blue for a two-band model system with a gap of width *E*_g_. (**c**) *β*(*E*_F_) values as a function of the HOMO–LUMO gap *E*g only.

The intrinsic conductance values of (DAD)_*n*_ polymers show that these are among the best molecular wires obtained so far for tunnelling transport. Comparing the intrinsic conductance values obtained in calculations and non-statistical single-molecule pulling experiments^12^,^18^,^19^ for a subset of representative chemical structures, all composed of connected or fused aromatic units, shows that (DAD)_*n*_ exhibits the smallest *β* value (Fig. 5). This reveals that a high conductance can even be achieved if the electronic delocalization along a molecular wire is very small and—as shown here—if the polymer exhibits a substantial flexibility, which is further evidenced by the formation of the DAD rings (Fig. 1f,g). At first glance, this might appear contradictory, but the increase in the effective mass m* because of the HOMO and LUMO localization is largely compensated by the HOMO–LUMO gap reduction, which is induced by the acceptor–donor alternation scheme (Supplementary Fig. 12).

Furthermore, we find good agreement between the calculated and experimental values as well as the universal curve^43^, which confirms the potential of this curve also for other molecular systems. Thus, by knowing m* for a given chemical structure from independent complex band structure calculations (as done in Fig. 5a), one can give a precise relationship between *E*_g_ and the inverse decay length *β.* Hence, the results in Fig. 5 emphasize the importance of both *E*_g_ and m* for the chemical design of the next generation of molecular wires^43^.

In order to underline this result, the inverse decay lengths *β* are plotted in Fig. 5c for comparison as a function of the HOMO–LUMO gap *E*_g_ only. The lack of a clear tendency is obvious as well as the difference between calculated and experimental values. Hence, by plotting the conductance property *β* as a function of the energy gap, predictions for other molecular structures can hardly be made—in strong contrast to the universal curve in Fig. 5a where the good agreement directly derives from the use of m* and the trend of *β* can be forecasted.

We believe that the alternation of donor and acceptor groups is the key for the high conductance since a homogeneous polymer that consists of thiophene units only (that is, the donor groups in the DAD chains) reveals a much larger band gap (Supplementary Fig. 12) and a reduced conductance (#2 in Fig. 5). Most importantly, electronic delocalization along a molecular wire is not necessarily required to obtain a rather good conductance in the absence of electronic states at the Fermi level. We believe that our results will stimulate the design of new polymers on the basis of donor and acceptor units that exhibit predictable current decay and considerable chain flexibility, both of which should be of interest for light-weight, bendable electronic applications.

## Methods

### Experiments

Measurements were performed under ultrahigh vacuum conditions with a low-temperature STM (Createc) operated at a temperature of 10 K. STS was performed with a lock-in amplifier (24 mV peak-to-peak modulation amplitude at 630 Hz frequency). STM images and d*I*/d*V* conductance maps were recorded in constant-current mode with the bias voltage referring to the sample with respect to the STM tip. The Au(111) sample was cleaned by repeated Ne ion sputtering (*E*=1.5 keV) and subsequent annealing up to 720 K. Molecules were evaporated from a Knudsen cell (at 445 K) on the clean Au(111) sample (at room temperature). Oligomers were produced from molecular building blocks via on-surface polymerization^36^, which is thermally induced by heating the sample (in contrast to polymerization with the STM tip^49^) at a typical temperature of 523 K for 10 min and subsequent covalent linking of the molecules.

### Calculations

The calculations of constant-current STM images and of electron transport properties through a molecular wire were carried out with the elastic scattering quantum chemistry technique^50^ using a semi-empirical Hamiltonian re-parameterized to describe the donor and acceptor monoelectronic states at the LUMO side of the monoelectronic molecular wire spectra. In elastic scattering quantum chemistry, the multichannel scattering matrix is exactly calculated on the tunnelling junction described by its electrodes, the molecule and the tip apex (in the STM configuration). The complete Hamiltonian of this tunnel junction is calculated for each atomic configuration of the tunnel junction: STM scanning, pulling or planar. Adsorbed on the Au(111) surface, the optimizations of the DAD molecular wire for image calculations and in a pulling junction configuration (connected to the tip on one side and to a non-reconstructed Au(111) surface on the other side) were performed with the semi-empirical ASED+ method^51^. For each tip height, the positions of all atoms along the molecular wire were optimized until a threshold of 0.01 eV Å^−1^ was reached on the force for each atom. Positions of the atoms in the tip and on the surface were maintained fixed during this optimization. The interaction of the molecular wire with the Au(111) surface was described by means of van der Waals interactions^51^, while the interaction of the end of the molecular wire with the tip was described by an ASED-optimized chemical bond. The parameters used in the calculations are presented in Supplementary Data 1–4 and described in the Supplementary Information.

### Synthesis

The monomer bis(5-bromo-2-thienyl)-benzobis(1,2,5-thiadiazole), referred to as the Br-DAD-Br monomer, was synthesized in a linear sequence of six steps (Supplementary Fig. 14) starting from commercial benzothiadiazole adapting protocols previously described in the literature^52^,^53^.

## Additional information

**How to cite this article**: Nacci, C. *et al.* Conductance of a single flexible molecular wire composed of alternating donor and acceptor units. *Nat. Commun.* 6:7397 doi: 10.1038/ncomms8397 (2015).

## Supplementary Material

Supplementary Figures, Supplementary Notes and Supplementary References Supplementary Figures 1-14, Supplementary Note 1 and Supplementary References

Supplementary Data 1 Atomic coordinates (x,y,z where z is the distance from the surface) used for STM image calculations of DAD (presented in Supplementary Fig.8).

Supplementary Data 2 Atomic coordinates (x,y,z where z is the distance from the surface) used for STM image calculations of the dimer (DAD)2 (presented in Supplementary Fig.9).

Supplementary Data 3 Atomic coordinates (x,y,z where z is the distance from the surface) used for STM image calculations of an oligomer (DAD)10 and the electronic transport calculations through (DAD)10 (Supplementary Fig.11).

Supplementary Data 4 Slater atomic orbitals parameters used in ESQC calculations.
