# Supplementary material for: Conductance of a single flexible molecular wire composed of alternating donor and acceptor units
Source: Nat Commun. 2015 Jul 6;6:7397. doi: 10.1038/ncomms8397 (PMC4507002; doi:10.1038/ncomms8397)
Supplement: Supplementary Figures, Supplementary Notes and Supplementary References — Supplementary Figures 1-14, Supplementary Note 1 and Supplementary References [file ncomms8397-s1.pdf]

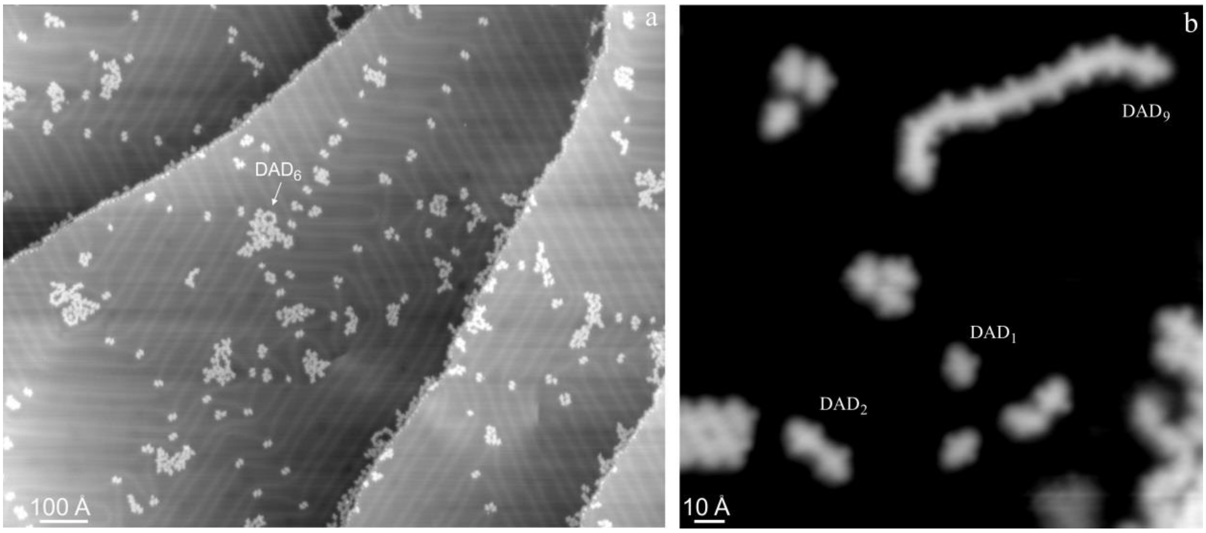

Supplementary Figure 1: *Constant-current STM images overview at large (a, 1373 Å × 1086 Å, 0.1 V, 100 pA) and small scale (b, 172 Å × 172 Å, 0.5 V, 100 pA) after dehalogenating the DAD species on Au(111) by thermal annealing up to 250 °C for five minutes. Single activated DAD monomers and dimers are present as well as a long DAD chain comprised of nine units (b).*

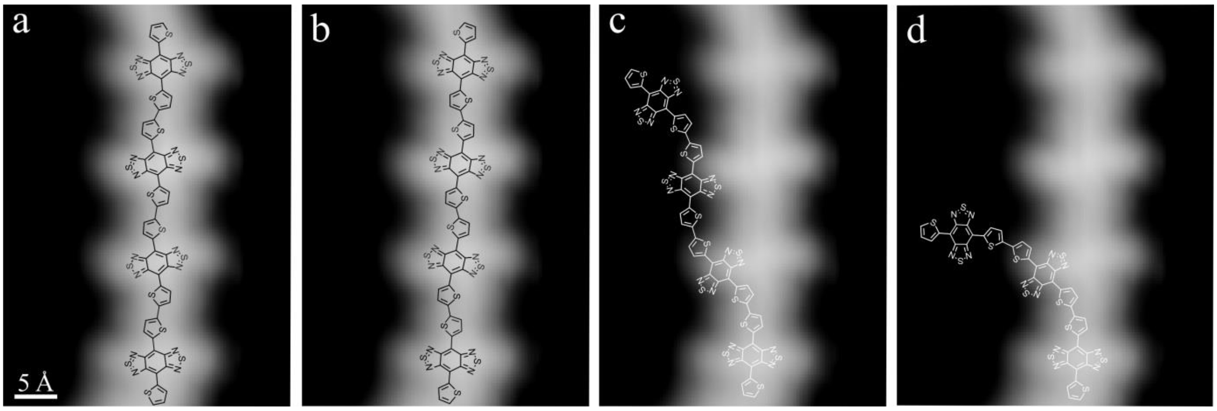

Supplementary Figure 2: *Determination of the S atom position by geometrical considerations with the same constant-current STM image of a straight segment of DAD wire (1 V, 50 pA). Four possible chemical structures are superimposed in (a-d). (a) The chemical structure of the wire has its inner bithiophene connections in a trans-configuration, (b) mirror-reflection of the chemical structure. (c-d) The inner bithiophene connections are arranged in a cis-configuration.*

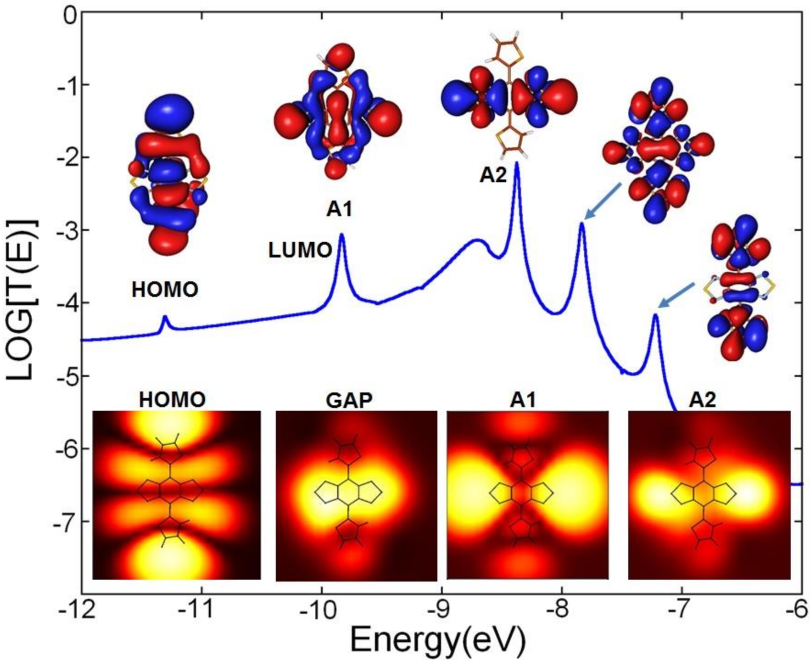

Supplementary Figure 3: *Calculated dI/dV electronic tunneling spectrum for the DAD monomer adsorbed flat on the Au(111) surface and represented in unit of $2e^2/h$ as a function of the electronic energy of the incident electron on the tunneling junction. A_1 is the LUMO resonance and A_2 the LUMO+1 resonance. All insets are constant-current dI/dV images calculated exactly at resonance (for the gap image, exactly at the middle of the HOMO-LUMO gap) at a fixed junction conductance of 1 GΩ. The height corrugations are 0.25 nm at the HOMO resonance, 0.035 nm in the gap, 0.35 nm at the A_1 resonance and 0.1 nm for the A_2 resonance. The bump left to the A_2 resonance is caused by a tip state.*

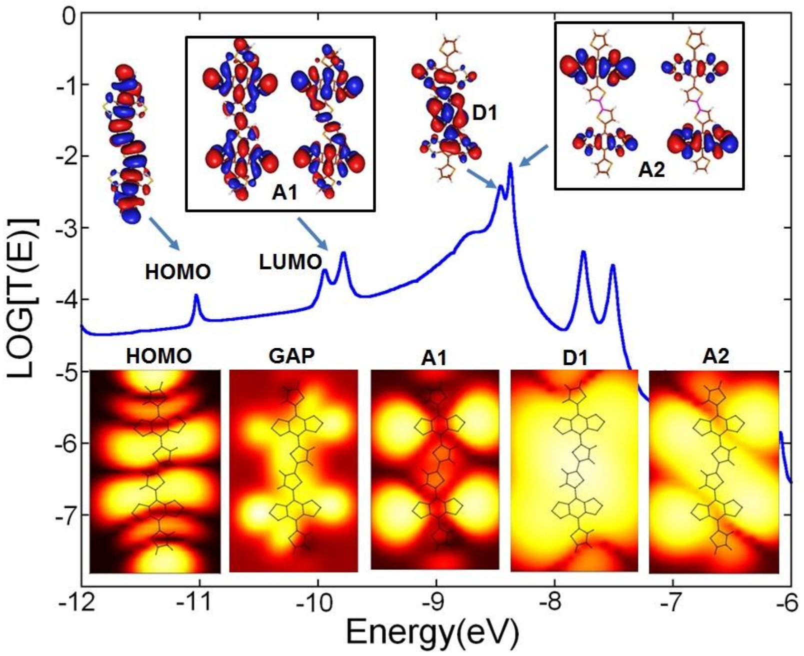

Supplementary Figure 4: *Calculated dI/dV electronic tunneling spectrum for the DAD dimer adsorbed flat on the Au(111) surface and represented in unit of $2e^2/h$ as a function of the electronic energy of the incident electron on the tunneling junction (The Fermi level is located around -10.5 eV for this semi-empirical calculation with only the valence electrons considered in ESQC). A_1 is the LUMO resonance doublet, D_1 is the new LUMO+1 state due to the dimer and A_2 is now a doubly degenerated LUMO+2 resonance. All insets are constant-current dI/dV images calculated exactly at resonance (for the gap image, exactly at the middle of the HOMO-LUMO gap) at a fixed junction conductance of 1 GΩ. The height corrugations are 0.25 nm at the HOMO resonance, 0.07 nm in the gap, 0.3 nm at the A_1 resonance and 0.7 n m for the D_1 resonance and 0.45 nm for the A_2 resonance. The shoulder left to the D_1 resonance is still coming from a tip state.*

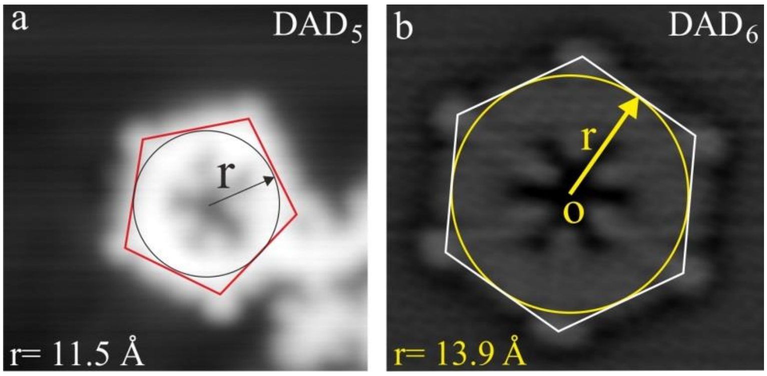

Supplementary Figure 5: *DAD-based closed structures. (a) Closed structures comprised of five molecules are found (86 Å × 86 Å, 0.5 V, 100 pA). (b) Laplace-filtered STM image of a DAD$_6$ ring (43.2 Å × 42 Å, 1 V, 50 pA). A curvature radius of around 13.9 ± 0.2 Å is determined for a DAD$_6$ ring and 11.5 ± 0.2 Å for a DAD$_5$ ring.*

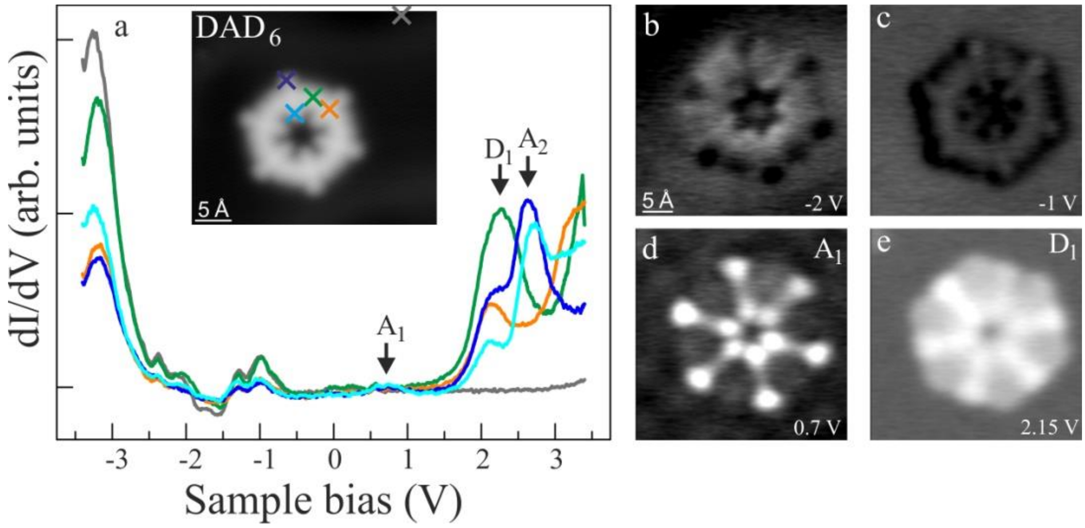

Supplementary Figure 6: *a) Constant-height dI/dV spectroscopy on a DAD$_6$ ring. Donor (D$_1$) and acceptor (A$_1$ and A$_2$) spectroscopic features are identified as previously seen in the case of open structures. Inset: constant-current STM image of DAD$_6$ ring (69 Å × 60 Å, 1 V , 50 pA). (b-e) Constant-current dI/dV maps (50 Å × 50 Å) of the DAD$_6$ density of states at energies of -2 V (b), -1 V (c), 0.7 V (d), 2.15 V (e).*

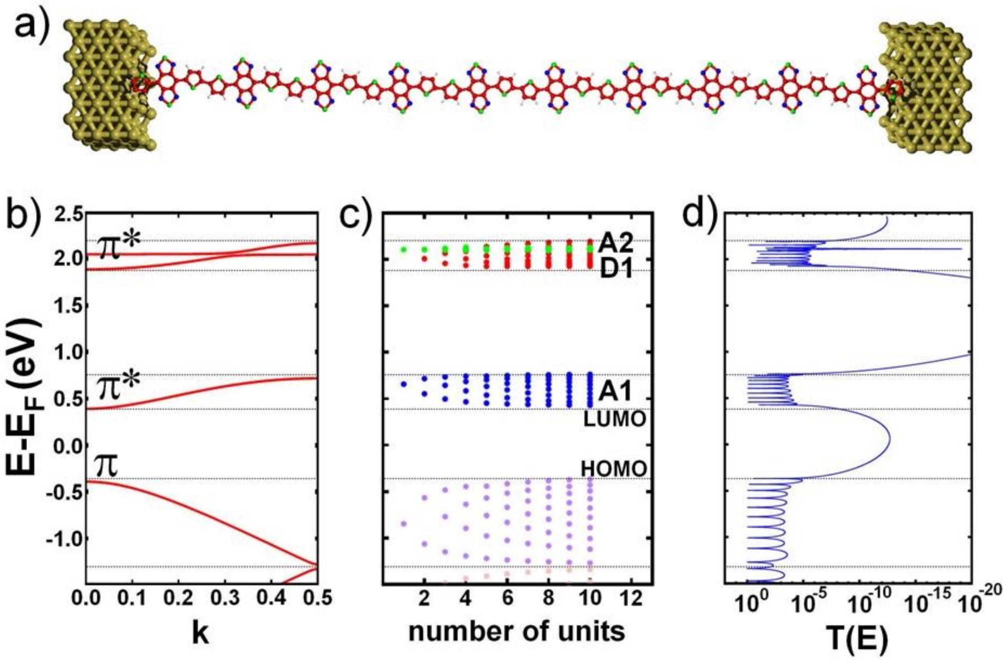

Supplementary Figure 7: *The electronic band structure (b), the positioning of the mono-electronic states as a function of the number of units (c) and the electronic transmission coefficient trough a 10 unit DAD molecular wires (d) all positioned at the same energy range around the HOMO-LUMO gap. On (d) the destructive interference when tunneling through the A_1 and D_1 states is clearly visible. The Fermi level is located around -10.5 eV for this semi-empirical calculation with only the valence electrons considered in ESQC. (a) is the atomic structure of the metal-(DAD)$_n$-metal molecular junction used to calculate the (d) T(E) presented here with a (DAD)$_{10}$ molecular wire.*

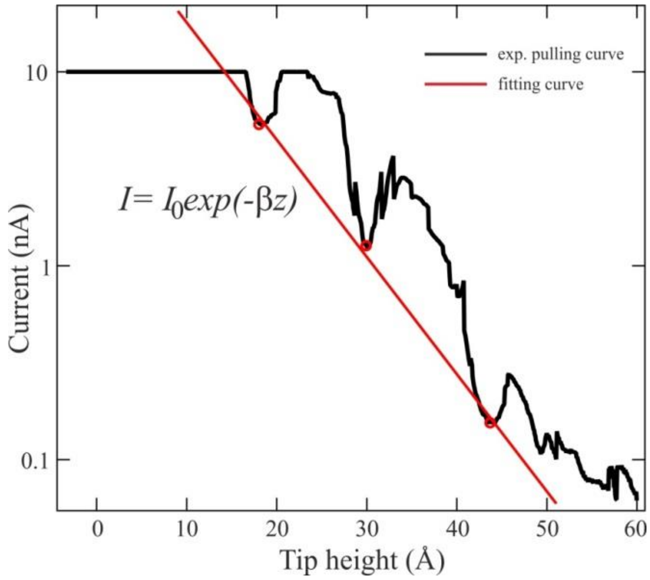

Supplementary Figure 8: *I(z) Pulling curve (black) taken at -100 mV (β= 0.24±0.05 Å^{-1}). Tunnel current oscillations are superimposed over the exponential current decay. The β current decay factor is evaluated by fitting the oscillation minima of the I(z) curve (red empty circles).*

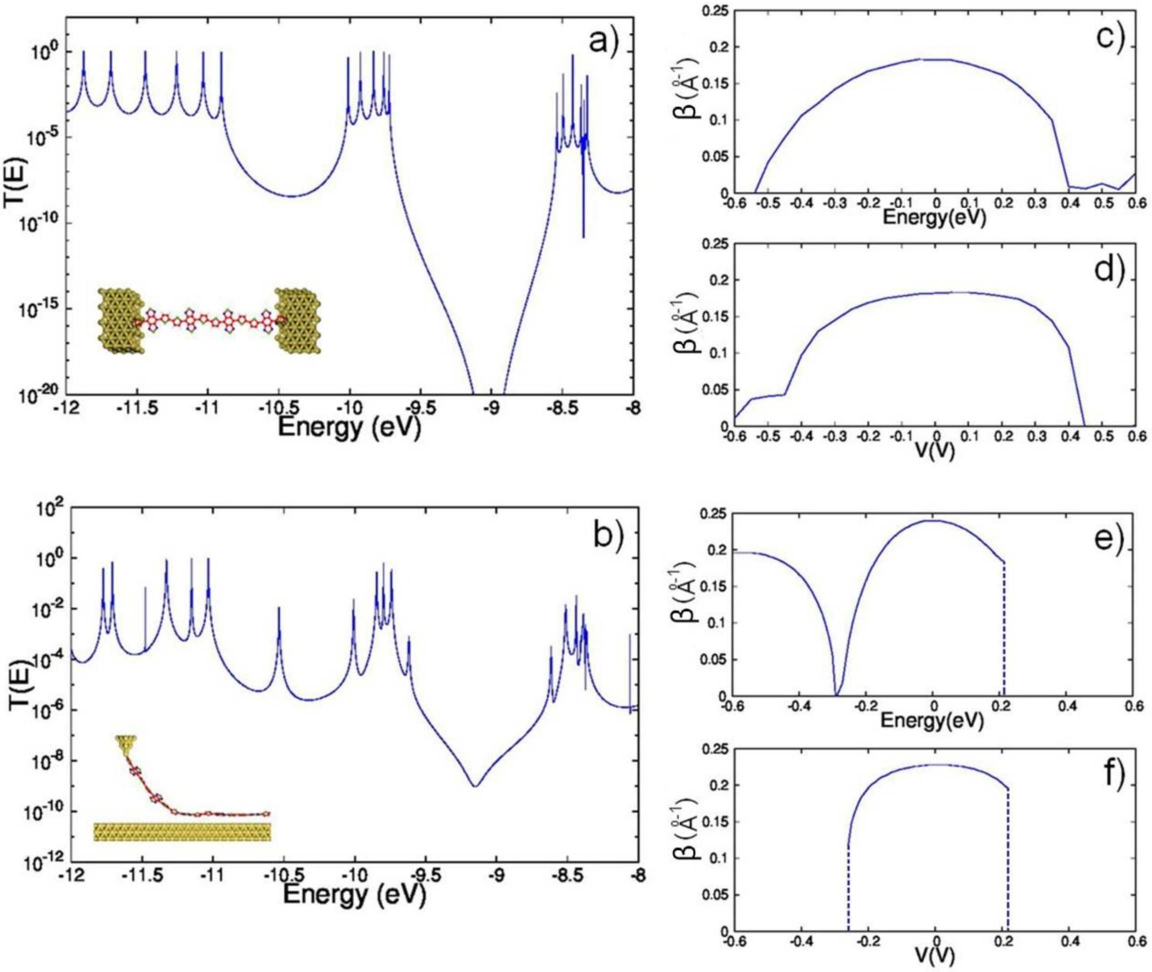

Supplementary Figure 9: *Transmission spectra for a planar junction between two electrodes (a) and (b) for a STM junction with an optimized conformation DAD chain in a pulling configuration. Notice the deformation of the pulled DAD wire T(E) spectrum due to the mechanical deformation of the molecular wire during the pulling. (c) and (e): the variation of the inverse decay length as a function of the reference energy (rescaled here for a Fermi level positioned at -10.5 eV) for the planar (a) and pulled (b) configuration. (d) and (f) the same curve respectively after calculating the full tunneling current intensity as a function of the bias voltage. Notice that for (e) and (f), the presence of the resonances in the center of the HOMO-LUMO gap due to the mechanical deformation of the molecular wire under pulling limits the calculation of the inverse decay length to a small energy (voltage) range. This has the effect to limit the measurable variation of the inverse decay length as a function of voltage (f) as compared to (d).*

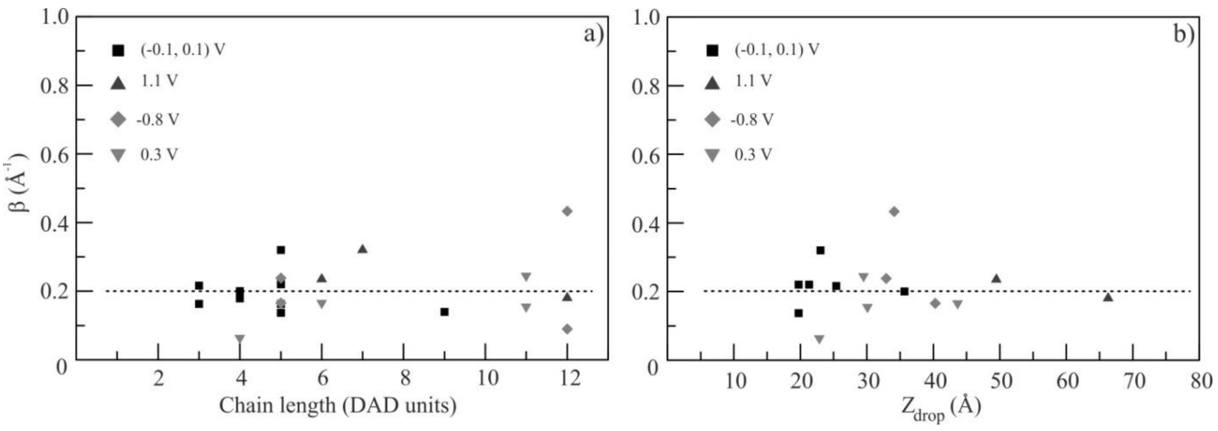

Supplementary Figure 10: *β values for (a) different oligomer lengths and (b) for different* Z_{drop} *values, i.e. the tip height at which the tip-oligomer contact breaks.*

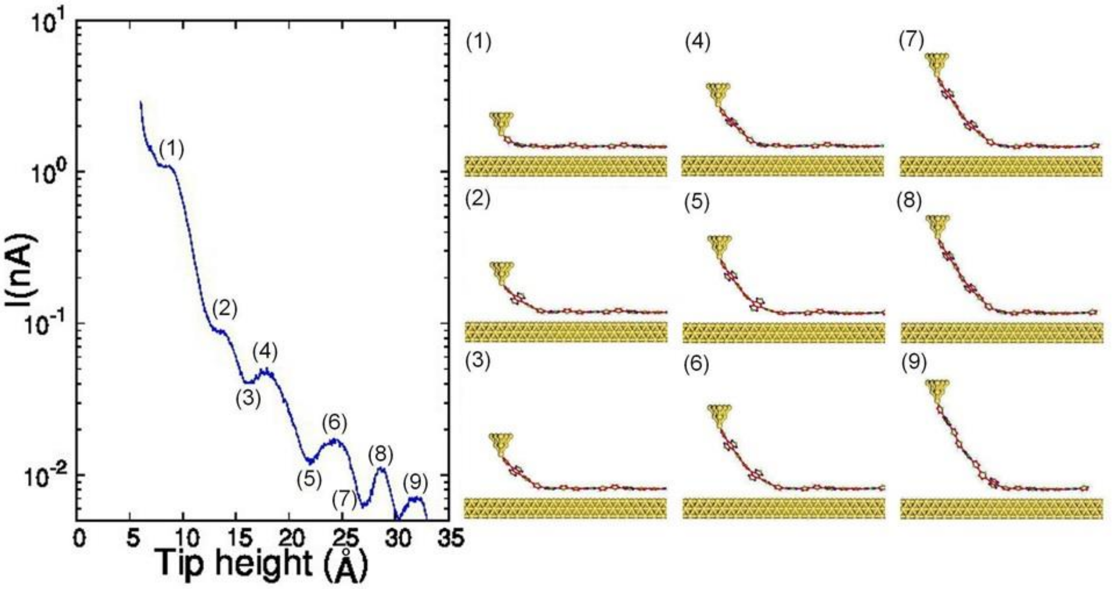

Supplementary Figure 11: *Calculated current in nA as a function of the tip height for the pulling of a* $(DAD)_5$ *oligomer for a bias voltage of 0.1 V together with the corresponding structures.*

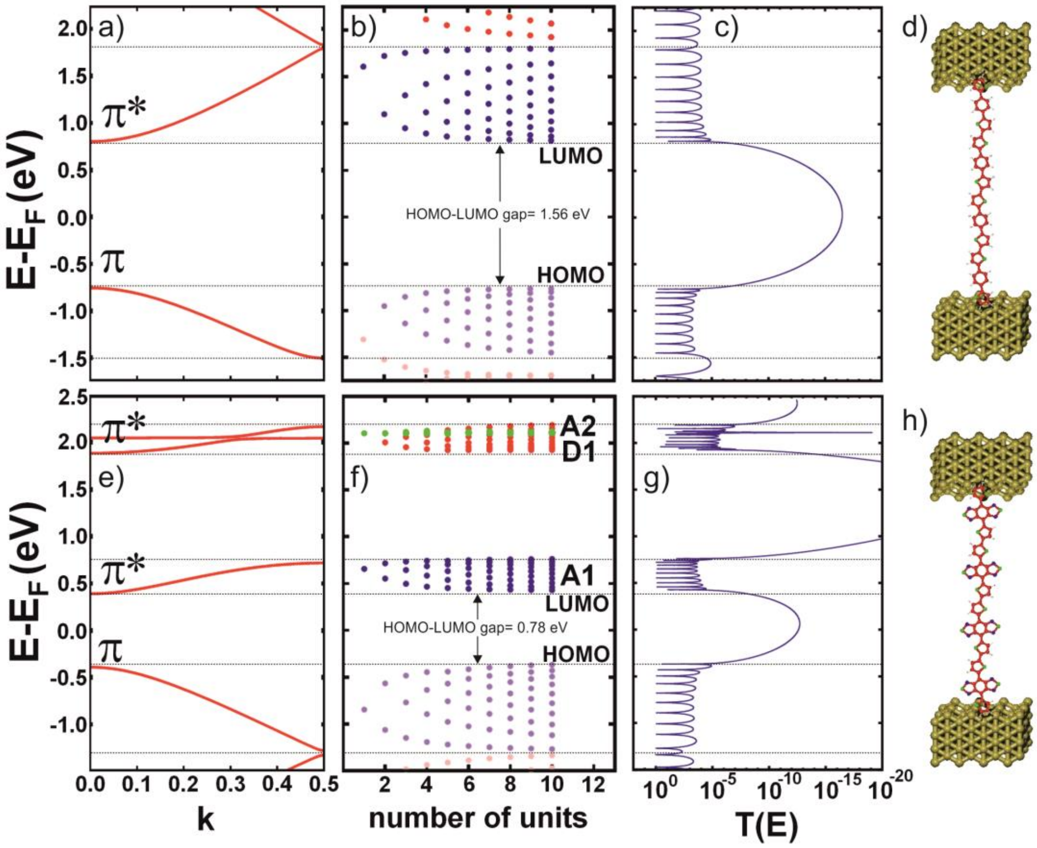

Supplementary Figure 12: *Tight-binding band structure and electron transmission coefficient for poly(dithiophene-phenyl) (a-d) and DAD molecular wires (e-h), respectively. Calculations has been done for long oligomers, i.e. ten units long each. Adding acceptor-withdrawing groups (DAD wires) induces a HOMO-LUMO gap reduction down to 0.78 eV, and a subtle modification of the conduction band structure splitted into less dispersive sub-bands* A_1 *and* D_1 *(*$\Delta E(A_1)$*: 0.40 eV,* $\Delta E(D_1)$*: 0.25 eV, no dispersion for sub-band* A_2*,* ΔE*(HOMO): 0.40 eV).*

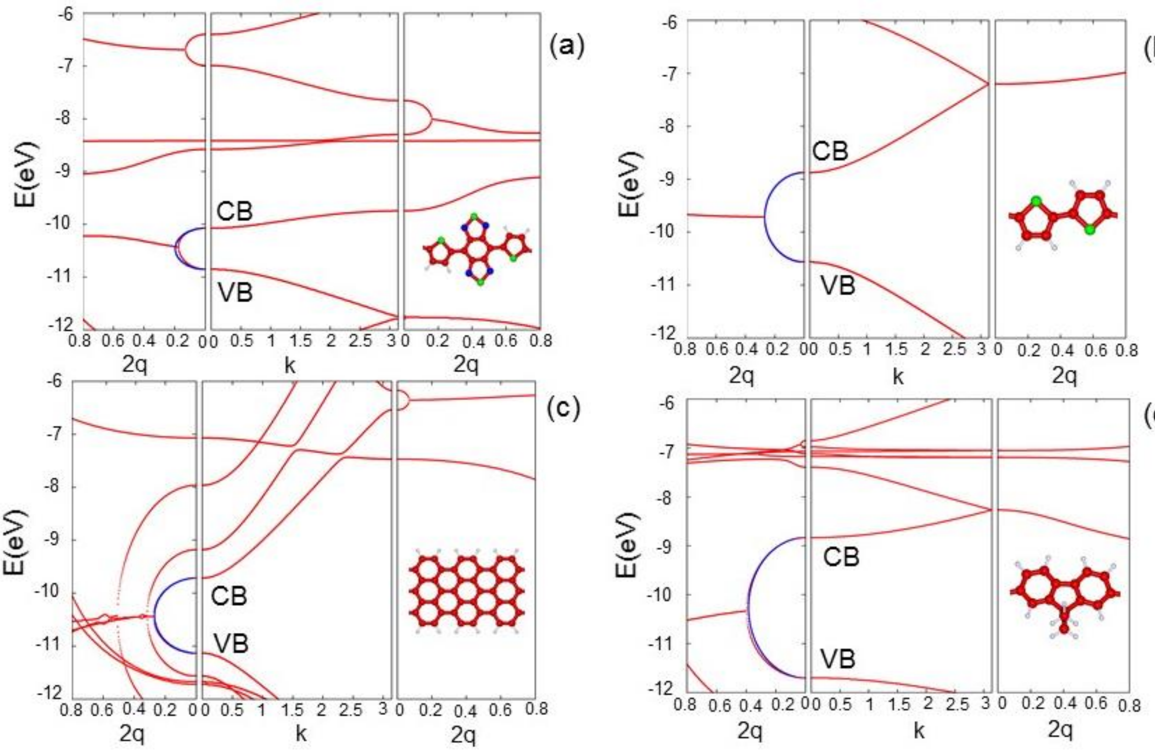

Supplementary Figure 13: *The complex valued band structures of four molecular wires used in Fig. 5 (DAD (a), thiophene (b), GNR (c) and DBTF (d)). The abscise scale was taken to be 2q to respect the real space characteristic of the tunneling decay through the molecular wire. The k scale is in unit of π. The effective mass m* is calculated at the zero of the second order derivative of the paraboloid. On each of them, the blue paraboloid corresponds to the universal* β(E) *tunneling decay law calculated for a two bands model using the m* obtained by the second derivative of the E(q) (red curve) in between the CB and VB band structures.*

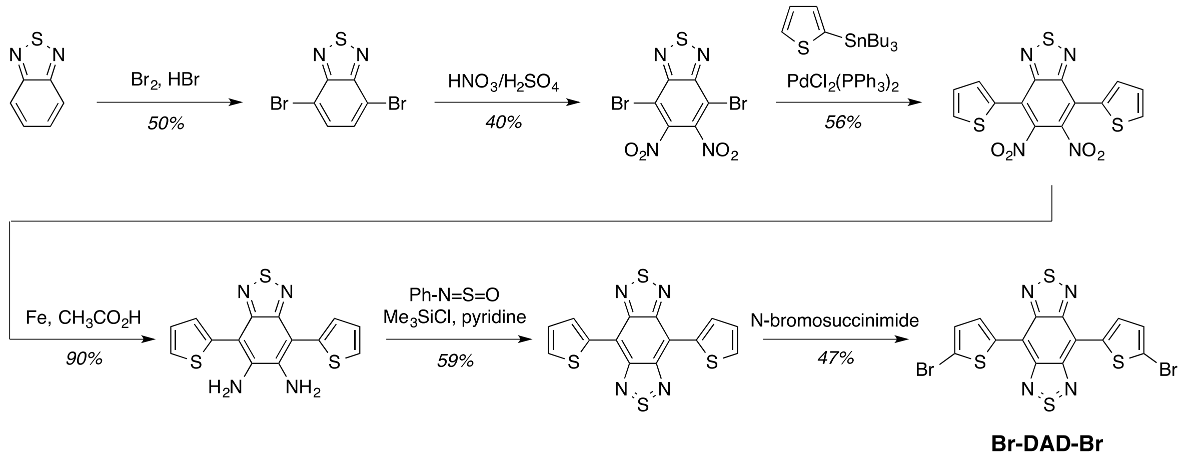

Supplementary Figure 14*: Chemical synthesis of the Br-DAD-Br monomer.*

Supplementary Note 1

Overview STM images

Supplementary Fig.1 shows a typical overview after dehalogenating DAD molecular species by thermal annealing (see Methods). A large variety of constituents is found: from single activated DAD monomers to covalently-linked chain with different length (two exemplars with two and nine units are clearly visible in Supplementary Fig.1b) or closed structures as DAD_6 ring (Supplementary Fig.1a).

Determination of the polymer conformation

The atomic positions in a molecule cannot be easily inferred from STM images and a precise determination requires to combine STM imaging and ESQC calculations [1]. However, some general informations on the molecular conformations can be obtained from measured distances and symmetry arguments when analyzing an STM image, as for instance for a DAD polymer: While the positions of the S atoms in the outer thiophene of a wire (endchains) remain ambiguous, the thiophene arrangement in the inner donor sites (bithiophene connections) can be determined by superimposing various chemical structures to the STM image (Supplementary Fig.2a-d).

As an example, a straight segment of DAD chain is shown in Supplementary Fig.2a and the relative chemical structure with bithiophene in *trans*-configuration adapts fairly well to the STM image. Importantly, the other possible *trans*-arrangement of inner thiophenes (mirror-

reflection of (a)) match less with the STM image (see the relative orientation of the acceptor sites compared to the bright lobes in the STM image). For completeness two other possibilities are plotted in Supplementary Fig.2, having chemical structures with the inner bithiophene connections in a *cis*-configuration (c-d). Notably, these *cis* arrangements do not allow to reproduce the imaged segment of wire.

Calculated electronic structure of a DAD monomer and $(DAD)_2$ dimer

The molecular orbitals of a DAD monomer, the electron transmission spectrum (with the tip apex located on a lateral thiadiazole group) and four characteristic ESQC images were calculated for the different electronic resonances as presented in Supplementary Fig.3 (the Fermi level is located around -10.5 eV for this semi-empirical calculation with only the valence electrons considered in ESQC). The HOMO resonance is extremely weak, indicating a small coupling of this electronic state with the Au(111) surface and explaining the difficulty to experimentally detect it by STM (see Fig.2 in the main text).

The A_1 and A_2 resonances derive from the central benzobis(thiadiazole) unit with a large STM junction conductance through a given thiadiazole group. The A_1 to A_2 resonance separation of 1.6 eV results from the large electronic interactions between the two thiadiazole π systems through the central phenyl π system. The two resonances above A_2 are characteristics of the LUMO+2 and LUMO+3 electronic states of the DAD monomer described in a mono-electronic approximation.

Calculations were also performed for a $(DAD)_2$ dimer (Supplementary Fig.4). The HOMO resonance is still very weak as compared to the LUMO ones. The A_1 and A_2 resonances are almost at the same energy position as compared with the DAD monomer indicative of a weak electronic interaction through the central thiophene dimer. A new D_1 resonance is now nicely appearing in between. It results from the bonding states of the two thiophenes composing now the central thiophene dimers. The calculated STM image at the A_1 state is very characteristic with its four large lobes. The D_1 molecular state is now very well located on the central thiophene dimer part of the $(DAD)_2$ molecule. It will give rise to the D_1 second conduction band of the $(DAD)_n$ polymer. Notice the nice doublet structure of the A_1 states with a splitting of about 0.1 eV. There is also a doublet structure for the A_2 states (see below) but corresponding to a degenerate LUMO+1 mono-electronic state with its two corresponding molecular orbitals.

Flexibility of the DAD polymers

The presence of bent open structures and closed DAD rings testify the high degree of intermolecular flexibility of DAD-based structures. The most abundant closed structure is

represented from DAD_6 ring (Supplementary Fig.5b) that is 20 times more abundant than DAD_5 (Supplementary Fig.5a). Their curvature radii are approximately 14 Å and 11.5 Å, respectively.

Tunneling spectroscopy of a DAD ring

We identified donor-like and acceptor-like electronic states on closed structures too. As reported for a DAD_6 ring in Supplementary Fig.6a, dI/dV spectroscopy reveals again the electronic states $A_{1,2}$ and D_1 roughly at the same energetic position as found for its linear counterpart (Fig.2 in the main text). dI/dV conductance mapping clearly shows that acceptor states A_1 (Supplementary Fig.6d) and A_2 (Fig.2q in the main text) have the same pattern with the signal maximum at the acceptor groups positions, while the donor state D_1 (Supplementary Fig.6e) reveal an inverted pattern as expected and as already seen for linear chains (Fig.2 in the main text). At negative bias voltages, the dI/dV spectroscopy does not reveal any molecular-related feature, and accordingly the conductance maps (Supplementary Fig.6b-c) are featureless.

Electronic destructive interference

Supplementary Fig.7 shows the electronic properties of the DAD polymer. The small dispersion of the A_1, D_1 bands and the zero dispersion of the A_2 band can be clearly seen. The calculated dispersion of the A_1 states is 0.4 eV for long $(DAD)_n$ oligomers. In the D_1, A_2 molecular orbital set, the D_1 states are dispersed on about a 0.25 eV range and the A_2 states not dispersed at all. There is a 1.3 eV calculated large separation between these two sets. Therefore the conduction band of a DAD polymer is composed of two not very dispersive sub-bands separated by a large gap.

The small 0.4 eV band dispersion of the A_1 conduction band correspond well to the 0.1 eV separation of the A_1 resonance doublet calculated for $(DAD)_2$. The D_1 band is much more dispersive than the A_2 one because of the through acceptor electronic interaction between two dithiophene units along the $(DAD)_n$ backbone. The HOMO state dispersion is about 0.8 eV which will give rise to a moderate 1 eV valence band for the DAD polymer. To confirm the measured dispersion of the $(DAD)_n$ unoccupied states presented in Fig.3 (main text) and above, band structure calculations of the $(DAD)_n$ polymer were performed, compared with the $(DAD)_n$ oligomer electronic states dispersion. Then, the ESQC electronic transmission coefficient through a $(DAD)_n$ oligomer with n =10 was calculated and compared with a (D-phenyl-D)$_n$ oligomer with n = 10 to identify the destructive interference through the $(DAD)_n$ oligomer between the A_1 and the D_1 states while tunneling in the energy range between those two conduction bands.

Fitting I(z) curves

The measured I(z) curves are not flat (in a semi-logarithmic plot), but exhibit characteristic oscillations (as presented in the main text). Such a behavior has already been observed for polyfluorene chains and has there – in combination with calculations – been assigned to the step-by-step detachment of the individual polymer units from the surface, thus the mechanical motion of the polymer [2].

Following this interpretation, fitting of the I(z) curve is done by using only the minima (or maxima) of the oscillation (see Supplementary Fig.8) and the fitting function $I = I_o \exp(-\beta z)$ (with I_o being the contact current and β the inverse decay length of the current through the wire). Note that irregular or noisy pulling curves, where the current oscillations cannot be clearly identified, have not been considered in the data analysis. When doing a statistical analysis with various I(z) curves of different oligomer pulling experiments, all done at similar small bias voltages (between -100 mV and +100 mV), we obtain an average value of β = 0.21 Å^{-1} with ±0.06 Å^{-1} statistical error.

Calculated β(E) and β(V) curves

Supplementary Fig.9 shows the calculated transmission spectra for the DAD chain in a planar junction between two electrodes and in a STM configuration where the molecule is connected between the tip and the surface.

Due to the bending of the molecule in the STM configuration, the gap of the chain is decreased in the STM junction and one state is pushed out of the valence band to the middle of the gap. This distortion of the electronic structure due to the bending of the molecule affects to the exponential decay of the transmission and the decay of the current as shown in Supplementary Fig.11. In the planar configuration between two electrodes, the exponential decay as a function of the energy, in the case of the transmission, and as a function of the voltage, in the case of the current, is almost planar in the middle of the gap. The decay decreases when reaching the valence and conduction band and it is zero in the band where the transmission is pseudo ballistic. The state in the middle of the gap affects drastically the decay curves because a pseudo ballistic transmission is produced at the resonance energy.

Inverse decay length β for different oligomer lengths

The β decay factors determined within the investigated range of bias voltages (from -0.8 up to +1.1 V) scatter around the average value and do not reveal a clear tendency. Thus, they seem to be independent from the oligomer lengths and Z_{drop} values (as shown in Supplementary Fig.10).

Comparison with homogeneous molecular wires

To support our choice of alternating donor and acceptor groups further, we present in Supplementary Fig.12 a comparison with long poly(dithiophene-phenyl) wires, reflecting simply the backbone of DAD molecular wires. Adding lateral electron-acceptor groups (thiadiazole groups) to the poly(dithiophene-phenyl) (i.e. changing from Supplementary Fig.12d to h) reveals a drastic change in terms of electronic structure.

A substantial shrinking of the HOMO-LUMO gap is predicted from calculations in favour of DAD wires (from 1.56 eV to 0.78 eV) with a clear benefit in terms of reduction of the inverse decay length β from 0.27 $Å^{-1}$ (poly(dithiophene-phenyl)) down to 0.21 $Å^{-1}$ (DAD, all in a planar configuration). A large dispersive conduction band is calculated in the case of the poly(dithiophene-phenyl) wire (Supplementary Fig.12a), while this band splits for DAD wires, resulting in the presence of two not very dispersive sub-bands A_1 and D_1 as shown in Supplementary Fig.12e-f separated by a large gap of 1.3 eV. Calculated electron transmission coefficients in a planar configuration through both wires (Supplementary Fig.12c and g) clearly reveal the destructive interference between the A_1 and D_1 states while tunneling in the energy range between those two conduction sub-bands. This peculiar electronic states dispersion with a large gap between the A_1 and the D_1 states affects β(E) via the m*(E) effective mass of tunneling electrons [3] confirming that β(E) is not only controlled by the HOMO-LUMO gap but also by the curvature of the β(E) inverse parabola.

Calculation of the effective mass m*

According to the electronic scattering theory applied to a periodic system (presenting an electronic band structure with gaps separating the bands), the mono-electronic Schrödinger equation can be solved for this periodic system assuming a real k vector or an imaginary k = iq vector (q in Supplementary Fig.13)[4]. For the real k, this gives rise to the standard E(k) band structure calculations, well-characterized in terms of scattering through the periodic system by a perfect T(E) transmission coefficient (for each real k vector E(k) band energy range).

For an imaginary k vector and for example between two bands, conduction band (CB) and valence band (VB), where in the corresponding energy range T(E) = 1, the dispersion relation has a parabolic form (as identified first by L. Brillouin for a genuine periodic system[5]). Generalization from semiconductor quantum transport calculations (in the valence-conduction band gap) to molecular wires has been reported[6] and can now also be calculated by DFT methods for simple molecular wires with a minimum of hetero-atoms[7].

For molecular wires, the number of calculated complex valued band structures remains limited as compared to semiconductor and magnetic materials since up to now there was no experimental way to measure the tunneling decay in the HOMO-LUMO gap through the

molecular wire. In all these calculations, the effective m* mass is given by calculating the curvature of the imaginary paraboloid at its maximal extension[6]. In general, m* depends on the energy of the tunneling electrons[6]. But in many cases and for molecular wires with not too many hetero-atoms per unit cell, m* calculated exactly at the saddle point of the tunneling paraboloid (and kept constant) is enough to reproduce the entire parabola using the universal law for the tunneling decay:

$$\beta(E) = \sqrt{\frac{2m^*(E)}{\hbar^2}\frac{(E-E_h)(E_l-E)}{\chi}}$$

Supplementary Equation 1

This is the case for example in the Supplementary Fig.13b, c and d band structures where in each case, the blue parabola was calculated using the above universal formula for β(E) with a constant m*. This approximation is generally working for quite symmetric valence and conduction band structure molecular wires[6]. The DAD molecular wire is an interesting exception with hetero-atoms because it presents an asymmetry between the CB and the VB leading to a small deviation between the calculated tunneling paraboloid and the universal decay law with an assumed constant m*. This is presented in Supplementary Fig.13a by superimposing the blue universal law on the calculated complex valued band structure. The direct consequence is that the paraboloid of the complex-valued part of the band structure is flatter at its saddle point as compared to the universal law, leading as a consequence to a smaller β value and therefore to a smaller m* for the same HOMO-LUMO gap. This is a direct consequence of the A_1, D_1 structure of the conduction band which is not present at the valence band side.

The fitting of the exact calculated paraboloid by the above β(E) universal law is possible by finding a good m*(E) function. But this type of work is out of the scope of this work and enters now the theory of complex valued band structure as explored already[6,7].

Calculation of the pulling mechanics

The mechanics of the pulling of a $(DAD)_5$ oligomer was studied theoretically by means of the semi-empirical ASED+ method[8]. The interaction of the $(DAD)_5$ oligomer with the Au(111) surface was described by means of van der Waals interactions[8]. As shown in previous works[2,3], when the tip is approached to the dangling bond of the molecule then a chemical bond is created between the molecule and the tip. After, the molecule is pulled up in steps of 0.05Å. For each step, the geometry of the molecule is optimized until a threshold of 0.01eV/Å

is reached for the force on each atom. For each step in the pulling, the electronic transmission between the tip and the electrode was computed by the ESQC technique[9].

The mechanics of the pulling reveals a rotation of the benzobis(thiadiazole) unit when this group is lifted up from the surface due to the van der Waals attraction. This rotation changes slightly the contact of the molecule with the surface making oscillations in the I(z) curve. This is in agreement with the experiments where oscillations of one unit size are observed. In the theoretical side, besides the 12 Å oscillation of a DAD unit, we obtain oscillations also for the benzobis(thiadiazole) and thiophene groups (Supplementary Fig.11). Maximum points in the curve correspond to configurations where the oligomer is completely parallel to the surface while minimums correspond to geometries where the part of the molecule lifted up is bent respect to the surface.

Atomic coordinates for DAD molecules

The atomic coordinates (x,y,z where z is the distance from the surface) used in the calculations are presented in separated files:

STM image calculations of DAD (Supplementary Data 1) presented in Supplementary Fig.8, of the dimer $(DAD)_2$ (Supplementary Data 2) presented in Supplementary Fig.9, for an oligomer $(DAD)_{10}$ (Supplementary Data 3) and the electronic transport calculations through $(DAD)_{10}$ (Supplementary Fig.11). For DAD and $(DAD)_2$, their molecular electronic structure on Au(111) were optimized using the semi-empirical ASED+ code.Furthermore, the Slater atomic orbitals parameters used in ESQC are presented (Supplementary Data 4).

Supplementary References

1. Sautet, P. & Joachim, C. Interpretation of STM images: copper-phthalocyanine on copper. *Surf. Sci.* **271**, 387-394 (1992).
2. Lafferentz, L. *et al.* Conductance of a single conjugated polymer as a continuous function of its length. *Science* **323**, 1193-1197 (2009).
3. Koch, M., Ample, F., Joachim, C. & Grill, L. Voltage-dependent conductance of a single graphene nanoribbon. *Nat. Nanotech.* **7**, 713-717 (2012).
4. Kohn, W. Analytic properties of Bloch waves and Wannier functions. *Phys. Rev.* **115**, 809 (1959).
5. Brillouin, L. & Parodi, M. Propagation des ondes dans les milieux periodiques. *Masson Paris* (1956).
6. Joachim, C. & Magoga, M. The effective mass of an electron when tunneling through a molecular wire. *Chem. Phys.* **281**, 347-352 (2002).
7. Ferretti, A. *et al. Ab initio* complex band structure of conjugated polymers: Effects of hybrid density functional theory and GW schemes. *Phys. Rev. B* **85**, 235105 (2012).
8. Ample, F. & Joachim, C. A semi-empirical study of polyacene molecules adsorbed on a Cu(110) surface. *Surf. Sci.* **600**, 3243-3251 (2006).

9 Sautet, P. & Joachim, C. Calculation of the benzene on rhodium STM images. *Chem. Phys. Lett.* **185**, 23-30 (1991).
